# A Predicting Nomogram for Mortality in Patients With COVID-19

**DOI:** 10.3389/fpubh.2020.00461

**Published:** 2020-08-11

**Authors:** Deng Pan, Dandan Cheng, Yiwei Cao, Chuan Hu, Fenglin Zou, Wencheng Yu, Tao Xu

**Affiliations:** ^1^Department of Pulmonary and Critical Care Medicine, Affiliated Hospital of Qingdao University, Qingdao, China; ^2^Department of Hematology, Tongji Medical College, Tongji Hospital, Huazhong University of Science and Technology, Wuhan, China; ^3^Department of Joint Surgery, Affiliated Hospital of Qingdao University, Qingdao, China; ^4^Department of Biliary-Pancreatic Surgery, Tongji Medical College, Tongji Hospital, Huazhong University of Science and Technology, Wuhan, China

**Keywords:** nomogram, predict, mortality, COVID-19, patients

## Abstract

**Background:** The global COVID-19 epidemic remains severe, with the cumulative global death toll reaching more than 207,170 as of May 2, 2020 (1).

**Purpose:** Our research objective is to establish a reliable nomogram to predict mortality in COVID-19 patients. The nomogram can help us distinguish between patients who are at high risk of death and need close attention.

**Patients and Methods:** For the single-center retrospective study, we collected 21 cases of patients who died in the critical illness area of the Optical Valley Branch of Tongji Hospital, Huazhong University of Science and Technology, from February 9 to March 10. Additionally, we selected 99 patients discharged during this period for analysis. The nomogram was constructed to predict the mortality for COVID-19 patients using the primary group of 120 patients and was validated using an independent cohort of 84 patients. We used multivariable logistic regression analysis to construct the prediction model. The nomogram was evaluated for calibration, differentiation, and clinical usefulness.

**Results:** The predictors included in the nomogram were c-reactive protein, PaO_2_/FiO_2_, and cTnI. The areas under the curves of the nomogram were 0.988 (95% CI: 0.972–1.000) and 0.956 (95% CI, 0.874–1.000) in the primary and validation groups, respectively. Decision curve analysis suggests that the nomogram may have clinical usefulness.

**Conclusion:** This study provides a nomogram containing c-reactive protein, PaO_2_/FiO_2_, and cTnI that can be conveniently used to predict individual mortality in COVID-19 patients. Next, we will collect as many cases as possible from multiple centers to build a more reliable nomogram to predict mortality for COVID-19 patients.

## Introduction

Since the outbreak of COVID-19 in Wuhan, Hubei province, in December 2019, it has had a major impact on people's lives, health, and property safety. It has rapidly expanded to 34 provincial divisions of China (2–4). As of April 28, 2020, the total number of confirmed cases in Wuhan has reached 68,128, accounting for 80.75% of the total number of confirmed cases in China, and 4,512 people have died in Wuhan, accounting for 97.18% of the total number of deaths in China (5).

SARS-CoV-2 is a binuclear virus that has a wide clinical spectrum of infection (6–8). In the past 2 months, only a few studies have analyzed prognostic factors for death, with repeated emphasis on factors such as age, lymphocytosis, leukocytosis, and elevated ALT (7). Thus, some of these potential prognostic factors need further validation, and a scoring system that accurately predicts the possible mortality risk is needed. Nomograms are used for multiple indicators to diagnose or predict disease onset or progression, making prognostic model results easier to read. Therefore, nomograms have been gradually applied in medical research and clinical practice (9, 10).

Here, we randomly selected some patients with definite clinical outcome (death or discharge) who had been admitted to the critical care unit of the Optical Valley Branch of Tongji Hospital, Huazhong University of Science and Technology, as of March 10, 2020 (11). The aim of our study was to establish a nomogram that incorporated demographics, clinical characteristics, and laboratory results to predict individual mortality in COVID-19 patients.

## Materials and Methods

### Patient Selection

We selected COVID-19 patients admitted to the critical illness area of the Optical Valley Branch of Tongji Hospital, Huazhong University of Science and Technology, from February 9, 2020, to March 10, 2020.

Patients were enrolled in this cohort if they: (1) were more than 18 years old; (2) were diagnosed with COVID-19 by multiplex real-time RT-PCR; (3) had complete clinical records of medical history and laboratory results. Patients were excluded if they: (1) were suspected with COVID-19; (2) had incomplete clinical records of medical history and laboratory results.

### Study Variables

We collected demographics, comorbidities, routine laboratory tests, immunological indicators, radiological images, and inpatient treatments (12). For each patient, the baseline characters were screened: gender, age, leukocytes, platelet–lymphocyte ratio (PLR), neutrophil–lymphocyte ratio (NLR), Charlson Comorbidity Index (CCI), CURB-65 score, PaO_2_/FiO_2_, interleukin-1β (IL-1β), interleukin-2R (IL-2R), interleukin-6 (IL-6), interleukin-8 (IL-8), interleukin-10 (IL-10), tumor necrosis factor-α (TNF-α), cardiac troponin I (cTnI), brain natriuretic peptide (BNP), calcitonin, c-reactive protein (CRP), lactate dehydrogenase (LDH), d-dimer, fibrinogen, history of hypertension, heart failure, coronary heart disease (CHD), diabetes, cough, expectoration, diarrhea, shortness of breath, body temperature above 38°C, and X-ray or CT findings.

### Construction of the Nomogram

We used the chi-square test for univariate analysis. Multivariable regression models were developed by incorporating meaningful factors in univariate analyses. Univariate and multivariate regression analysis was used to analyze the risk factors in the primary cohort (13). Then we construct a nomogram on the basis of the multivariate logistic regression model.

### Statistical Analysis

Patients' demographic and clinical characteristics were analyzed using descriptive methods, with standard summary statistics including the median, interquartile range (IQR), and proportions. Categorical variables were processed by Chi-square or Fisher's exact tests, as appropriate. We tested the accuracy of the nomograms by discrimination and calibration both in the primary and the validation cohort. We used the receiver operating characteristic curve (ROC) to assess the discriminative ability of the nomogram and then assessed the area under the curve (AUC) (14, 15). Calibration curves were used to compare the association between actual outcomes and predicted probabilities (16). Decision curve analysis (DCA) was performed by calculating the net benefits for a range of threshold probabilities to evaluate the clinical utility of the nomogram (17, 18). Statistical analysis was performed using SPSS 23 (IBM, Chicago, IL, USA) and R version 3.4.4. All statistical tests were two-tailed, and a *P* < 0.05 was considered to be statistically significant.

## Results

### Patient Characteristics

Between February 9, 2020, and March 10, 2020, from more than 600 patients in the critical illness area of the Optical Valley Branch of Tongji Hospital, 120 cases were selected for inclusion in the study by means of a random number table. The average age of the 120 patients was 62.16 years old, and there were 50 females and 70 males (19). The majority of the patients were over 60 years old (69.20%). Among the 120 cases, 90 (75.0%) developed cough symptoms with or without expectoration, 62 (51.7%) had high fever (≥38°C), 68 (56.7%) had mild shortness of breath, and 15 (12.5%) developed diarrhea. The vast majority of patients exhibited bilateral invasion in chest X-ray and CT, mostly with a patchy and ground-glass appearance (Table 1).

**Table 1:** Results of the univariate association analyses.

**Characteristics**	**Death set**	**Survival set**	***Z*-value**	***P*-value**
Age, year	70 (66, 78.5)	64 (51, 70)	−3.273	0.001
Sex (male)	17	53	5.358	0.021
Sex (female)	4	46		
WBC	9.01 (5.42, 14.62)	5.47 (4.49, 6.81)	−3.398	0.001
NLR	10.44 (5.70, 25.10)	2.50 (1.80, 3.84)	−5.688	0.000
PLR	305.88 (143.23, 352.80)	204.40 (149.43, 312.73)	−1.433	0.152
PCT	0.29 (0.15, 0.57)	0.06 (0.05, 0.07)	−6.560	0.000
CRP	113.3 (63.55, 145.4)	8.9 (2.4, 32.4)	−5.888	0.000
LDH	566 (327.5, 667.5)	245 (190, 285)	−5.046	0.000
Fibrinogen	4.9 (3.22, 6.19)	5.18 (4.1, 5.85)	−0.597	0.550
PaO_2_/FiO_2_	105.43 (76.05, 161.98)	331.76 (233.89, 390.54)	−6.351	0.000
Serumcalcium(L)	19	77	1.043	0.307
TNI(H)	11	5	29.615	0.000
BNP(H)	18	23	30.070	0.000
CURB-65(0–1)	10	93	26.879	0.000
CURB-65(2–5)	11	6		
CCI index(0–2)	18	96	2.555	0.110
CCI index(3-)	3	3		
IL-1β(H)	4	16	0.000	1.000
IL-2R(H)	17	34	15.554	0.000
IL-6(H)	19	36	20.434	0.000
IL-8(H)	4	4	4.091	0.043
IL-10(H)	8	8	11.034	0.001
TNF-α(H)	16	45	6.549	0.010
D-dimer(H)	21	68	8.866	0.003
Hypertension	13	31	6.982	0.008
Heart failure	1	0		0.175
CHD	4	5	3.083	0.079
Diabetes	4	14	0.055	0.814
Cough	14	76	0.943	0.332
Expectoration	7	44	0.875	0.350
Diarrhea	2	13	0.008	0.928
≥38°C	11	51	0.005	0.943
GGO	8	30	0.486	0.486
Consolidation	0	13	1.883	0.170
Pathy	15	77	0.116	0.733
Fibrosis	0	3		1.000

### Independent Prognostic Factors

We used univariate and multivariate analyses to identify the prognostic factors (20, 21). The univariate analysis revealed that age (*p* = 0.001), Male/Female (*p* = 0.021), WBC (*p* = 0.001), NLR (*p* = 0.000), PCT (*p* = 0.000), CRP (*p* = 0.000), LDH (*p* = 0.000), PaO_2_/FiO_2_ (*p* = 0.000), Serum calcium (*p* = 0.307), cTnI (*p* = 0.000), BNP (*p* = 0.000), CURB-65 (*p* = 0.000), IL-2R (*p* = 0.000), IL-6 (*p* = 0.000), IL-8 (*p* = 0.043), IL-10 (*p* = 0.001), TNF-α (*p* = 0.01), D-dimer (*p* = 0.003), and Hypertension (*p* = 0.008) were significant prognostic factors. The multivariate analysis revealed that CRP (*p* = 0.004), PaO_2_/FiO_2_ (*p* = 0.002), and cTnI (*p* = 0.016) were independent prognostic factors for predicting the mortality of COVID-19 patients (22).

### Development and Validation of a Nomogram

**Figure 1:**
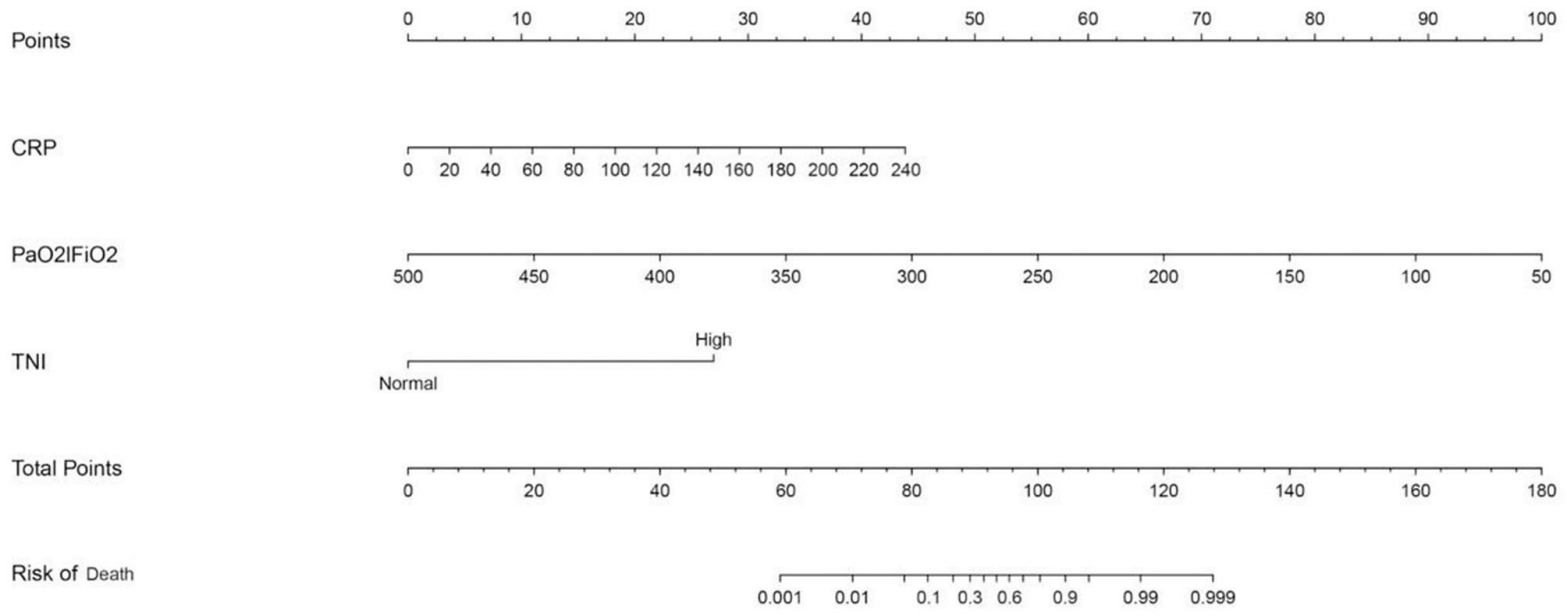
Nomogram predicting mortality for COVID-19 patients with three available factors: CRP, PaO_2_/FiO_2_, and cTnI.

Multivariate logistic regression analysis determined that CRP, PaO_2_/FiO_2_, and cTnI were independent predictors (Table 2). The model with three independent predictors is established and represented by a nomogram (Figure 1).

**Table 2:** Multivariate logistic regression of death in COVID-19 patients.

	**B**	**SE**	**Wald**	**OR(95%CI)**	***P*-value**
CRP	0.037	0.013	8.271	1.037 (1.012, 1.064)	0.004
PaO2/FiO2	−0.045	0.015	9.144	0.956 (0.929, 0.984)	0.002
TNI	5.417	2.244	5.830	225.296 (2.773, 18303.1)	0.016

We predicted the probability of death in patients with COVID-19 by calculating the sum of points in the nomogram corresponding to patient characteristics. Regarding the clinical application of this nomogram, we assumed that, if a patient's CRP was 20 mg/L, the PaO_2_/FiO_2_ was 350, and the cTnI was increased, then the corresponding score of this patient was 3+33+26, a total of 62 points, so the corresponding risk of death of this patient was between 0.001 and 0.01, and this patient was relatively safe.

**Figure 2:**
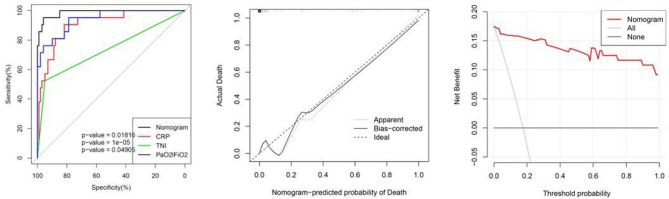
From left to right: ROC curves of the nomogram and the three factors; calibration curve of the nomogram in the cohort; decision curve analysis for the nomogram. The y-axis measures the net benefit. The red line represents the nomogram. The gray line represents the assumption that all patients have died. The thin black link represents the assumption that no patients have died. The net benefit was calculated by subtracting the proportion of all patients who are false positive from the proportion who are true positive, weighting by the relative harm of forgoing treatment compared with the negative consequences of unnecessary treatment.

The AUC for the prediction nomogram was 0.988, 95% CI, 0.972–1.000 (23). The AUC for CRP was 0.910. The AUC for PaO_2_/FiO_2_ was 0.942. The AUC for cTnI was 0.737. The nomogram decision curve analysis is shown in Figure 2. The calibration curve of the nomogram for the probability of mortality demonstrated good agreement between prediction and observation in the primary cohort (Figure 2) (24). The decision curve showed that if the threshold probability of a patient or doctor is >0%, using the nomogram to predict the mortality for COVID-19 patients adds more benefit. Within this range, net benefit was comparable, with several overlaps, on the basis of the nomogram (25).

**Figure 3:**
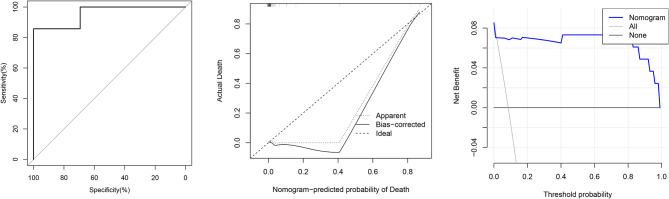
From left to right: ROC curves of the Nomogram and the three factors; calibration curve of the nomogram in the cohort; decision curve analysis for the nomogram.

Validation was performed by using another 84 COVID-19 patients. In the validation cohort, the independent risk factors included in the nomogram were examined. The AUC of the nomogram was 0.956 (95% CI: 0.874–1.000). Figure 3 shows that the calibration plot for the probability of mortality demonstrated good agreement between the prediction by nomogram and actual observation.

## Discussion

Since 2003, coronavirus has caused a number of major public health events (26, 27). Generally, it was not until the outbreak of severe acute respiratory syndrome (SARS) in Guangdong, China, in 2002 and 2003 that the coronavirus was found to be highly pathogenic to humans. Another highly pathogenic coronavirus is the Middle East respiratory syndrome (MERS) coronavirus, which emerged in 2012 in Middle Eastern countries (28–31). COVID-19 is another highly pathogenic coronavirus that has been added to human history. Although COVID-19 has a mortality rate of <3% compared to SARS (9.6%) and MERS (34%), its effects remain extremely sudden and devastating (32, 33).

Previous studies have shown that coronaviruses can cause respiratory and intestinal infections (34). According to recent reports, the most common symptoms of COVID-19 patients are fever, cough, myalgia, and fatigue, while the less common are expectoration, hemoptysis, and diarrhea (35–39). Most patients have mild symptoms and good prognosis. Mild patients may have only a low fever and mild weakness, with no obvious signs of pneumonia (40). In the late stage of infection, a variety of complications occur in critically ill patients, including ARDS, septic shock, refractory metabolic acidosis, acute myocardial injury, disseminated intravascular coagulation, and even death (5, 6). Therefore, early diagnosis and early assessment of the patient's prognosis are crucial for controlling the outbreak.

Corticosteroids were used extensively during the outbreaks of severe SARS and MERS and are being applied in COVID-19 patients. Corticosteroids suppress inflammation in the lungs but also inhibit immune responses and pathogen clearance at the same time (41). The clinical benefit of corticosteroids in COVID-19 needs further clinical trials to confirm (42, 43).

In previous studies, several prognostic models have been developed. For example, the NLR was the most effective prognostic factor affecting prognosis for severe COVID-19 patients (44).

In our study, this nomogram based on three variables, CRP, PaO_2_/FiO_2_, and cTnI, can provide a more accurate assessment and prediction of mortality at admission for COVID-19 patients. If patients with high mortality rates are properly and promptly identified, they should be more likely to benefit from nutritional support in nursing care and close attention in clinical care, which will ultimately have a positive impact on their recovery (45, 46).

The variables in the nomogram we constructed were easily accessible from routine clinical work. As a result, clinicians can use this simple, intuitive predictive tool to draw a few lines in a few seconds to make a quick assessment of a patient's prognosis with no computational difficulty. Additionally, our model can help clinical workers rationally allocate medical resources to reduce the fatality rate of COVID-19 when medical resources are scarce.

There are still some limitations to our study. First, the study was at a single center with a small sample, and the next step is to include as many cases as possible from multiple centers to build a more reliable predicting nomogram for mortality in COVID-19 patients. Second, we excluded patients whose case data were incomplete, which could have resulted in selection bias.

## Conclusions

To sum up, to better identify the prognosis of COVID-19 patients, identify severe patients as early as possible, improve the cure rate, and reduce the fatality rate, we constructed a nomogram based on 120 patients to predict mortality. The proposed nomogram considered three independent risk factors, namely, CRP, PaO_2_/FiO_2_, and cTnI. We have confirmed that this nomogram has excellent discrimination and clinical availability.

## Data Availability Statement

The raw data supporting the conclusions of this article will be made available by the authors, without undue reservation.

## Ethics Statement

The studies involving human participants were reviewed and approved by Medical ethics committee of affiliated hospital of Qingdao university. The ethics committee waived the requirement of written informed consent for participation. Written informed consent was obtained from the individual(s) for the publication of any potentially identifiable images or data included in this article.

## Author Contributions

DP: data analysis and visualization. DP, DC, YC, and CH: writing original draft. FZ, TX, and WY: review and editing. All authors contributed to the article and approved the submitted version.

## Conflict of Interest

The authors declare that the research was conducted in the absence of any commercial or financial relationships that could be construed as a potential conflict of interest.
